# COVID-19 severity, breakthrough infections and vaccine safety in young individuals with autoimmune diseases: insights from the COVAD study

**DOI:** 10.1007/s00296-024-05654-w

**Published:** 2024-07-13

**Authors:** Alessia Alunno, Francesco Carubbi, Ai Lyn Tan, Parikshit Sen, Lorenzo Cavagna, Mrudula Joshi, Jessica Day, Sreoshy Saha, Carlos Enrique Toro Gutiérrez, Carlo Vinicio Caballero-Uribe, Oliver Distler, Hector Chinoy, Rohit Aggarwal, Vikas Agarwal, Latika Gupta, COVAD Study Group, Elena Nikiphorou, Arvind Nune, James B. Lilleker, John D. Pauling, Chris Wincup, Armen Yuri Gasparyan, Vishwesh Agarwal, Esha Kadam, Bhupen Barman, Yogesh Preet Singh, Rajiv Ranjan, Avinash Jain, Sapan C. Pandya, Rakesh Kumar Pilania, Aman Sharma, Manesh Manoj M, Vikas Gupta, Chengappa G. Kavadichanda, Pradeepta Sekhar Patro, Sajal Ajmani, Sanat Phatak, Rudra Prosad Goswami, Abhra Chandra Chowdhury, Ashish Jacob Mathew, Padnamabha Shenoy, Ajay Asranna, Keerthi Talari Bommakanti, Anuj Shukla, Arunkumar R. Pande, Prithvi Sanjeevkumar Gaur, Mahabaleshwar Mamadapur, Akanksha Ghodke, Kunal Chandwar, Naitica Darooka, Praggya Yaadav, Babur Salim, Zoha Zahid Fazal, Mahnoor Javaid, Resit Yildrim, Ashima Makol, Tulika Chatterjee, Aarat Patel, Margherita Giannini, Francois Maurier, Julien Campagne, Alain Meyer, Nicoletta Del Papa, Gianluca Sambataro, Atzeni Fabiola, Marcello Govoni, Simone Parisi, Elena Bartoloni Bocci, Gian Domenico Sebastiani, Enrico Fusaro, Marco Sebastiani, Luca Quartuccio, Franco Franceschini, Pier Paolo Sainaghi, Giovanni Orsolini, Rossella De Angelis, Maria Giovanna Danielli, Vincenzo Venerito, Silvia Grignaschi, Alessandro Giollo, Laura Andreoli, Daniele Lini, Florenzo Iannone, Marco Fornaro, Lisa S. Traboco, Syahrul Sazliyana Shaharir, Chou Luan Tan, Suryo Anggoro Kusumo Wibowo, Abraham Edgar Gracia- Ramos, Miguel A. Saavedra, Erick Adrian Zamora Tehozol, Jorge Rojas Serrano, Ignacio García-De La Torre, Iris J. Colunga-Pedraza, Javier Merayo-Chalico, Raquel Aranega, Jesús Loarce-Martos, Sergio Prieto-González, Samuel Katsuyuki Shinjo, Leonardo Santos Hoff, Masataka Kuwana, Akira Yoshida, Ran Nakashima, Shinji Sato, Naoki Kimura, Yuko Kaneko, Takahisa Gono, Ioannis Parodis, Marcin Milchert, Johannes Knitza, Stylianos Tomaras, Fabian Nikolai Proft, Marie-Therese Holzer, Karen Schreiber, Margarita Aleksandrovna Gromova, Or Aharonov, Melinda Nagy-Vincze, Zoltán Griger, Nelly Ziade, Ihsane Hmamouchi, Pr Imane El bouchti, Zineb Baba, Dzifa Dey, Uyi Ima-Edomwonyi, Ibukunoluwa Dedeke, Emorinken Airenakho, Nwankwo Henry Madu, Abubakar Yerima, Hakeem Olaosebikan, Celestine Chibuzo Okwara, A Becky, Ouma Devi Koussougbo, Elisa Palalane, Daman Langguth, Vidya Limaye, Merrilee Needham, Nilesh Srivastav, Marie Hudson, Océane Landon-Cardinal, Tsvetelina Velikova, Russka Shumnalieva, Wilmer Gerardo Rojas Zuleta, Álvaro Arbeláez, Javier Cajas, José António Pereira Silva, João Eurico Fonseca, Olena Zimba, Ho So, Manuel Francisco Ugarte-Gil, Lyn Chinchay, José Proaño Bernaola, Victorio Pimentel, A. T. M. Tanveer Hasan, Binit Vaidya, Tamer A. Gheita, Hanan Mohamed Fathi, Reem Hamdy A. Mohammed, Yi-Ming Chen, Ghita Harifi, Lina El Kibbi, Hussein Halabi, Phonpen Akawatcharangura Goo, Wanruchada Katchamart, Yurilís Fuentes-Silva, Karoll Cabriza, Jonathan Losanto, Nelly Colaman, Antonio Cachafeiro-Vilar, Generoso Guerra Bautista, Enrique Julio Giraldo Ho, Lilith Stange Nunez, Cristian Vergara M, Jossiell Then Báez, Hugo Alonzo, Carlos Benito Santiago Pastelin, Rodrigo García Salinas, Alejandro Quiñónez Obiols, Nilmo Chávez, Andrea Bran Ordóñez, Gil Alberto Reyes Llerena, Radames Sierra-Zorita, Dina Arrieta, Eduardo Romero Hidalgo, Ricardo Saenz, Idania Escalante M, Wendy Calapaqui, Ivonne Quezada, Gabriela Arredond

**Affiliations:** 1 Internal Medicine and Nephrology Division, Department of Clinical Medicine, Life, Health and Environmental Sciences, University of L’Aquila and San Salvatore Hospital, Piazzale Salvatore Tommasi 1, 67100 L’Aquila, Italy; 2 NIHR Leeds Biomedical Research Centre, Leeds Teaching Hospitals Trust, Leeds, UK; 3 Leeds Institute of Rheumatic and Musculoskeletal Medicine, University of Leeds, Leeds, UK; 4 Maulana Azad Medical College, 2-Bahadurshah Zafar Marg, New Delhi, Delhi 110002 India; 5 Rheumatology Unit, Dipartimento di Medicine Interna e Terapia Medica, Università degli Studi di Pavia, Pavia, Lombardy Italy; 6 Byramjee Jeejeebhoy Government Medical College and Sassoon General Hospitals, Pune, India; 7 Department of Rheumatology, Royal Melbourne Hospital, Parkville, VIC 3050 Australia; 8 Walter and Eliza Hall Institute of Medical Research, Parkville, VIC 3052 Australia; 9 Department of Medical Biology, University of Melbourne, Parkville, VIC 3052 Australia; 10 Mymensingh Medical College, Mymensingh, Bangladesh; 11 Reference Center for Osteoporosis, Rheumatology and Dermatology, Pontifica Universidad Javeriana Cali, Cali, Colombia; 12 Department of Medicine, Hospital Universidad del Norte, Barranquilla, Atlantico Colombia; 13 Department of Rheumatology, University Hospital Zurich, University of Zurich, Zurich, Switzerland; 14 Division of Musculoskeletal and Dermatological Sciences, Centre for Musculoskeletal Research, School of Biological Sciences, Faculty of Biology, Medicine and Health, Manchester Academic Health Science Centre, The University of Manchester, Manchester, UK; 15 National Institute for Health Research Manchester Biomedical Research Centre, Manchester University NHS Foundation Trust, The University of Manchester, Manchester, UK; 16 Department of Rheumatology, Salford Royal Hospital, Northern Care Alliance NHS Foundation Trust, Salford, UK; 17 Division of Rheumatology and Clinical Immunology, University of Pittsburgh School of Medicine, Pittsburgh, PA USA; 18 Department of Clinical Immunology and Rheumatology, Sanjay Gandhi Postgraduate Institute of Medical Sciences, Lucknow, India; 19 Department of Rheumatology, Royal Wolverhampton Hospitals NHS Trust, Wolverhampton, UK; 20 Department of Rheumatology, City Hospital, Sandwell and West Birmingham Hospitals NHS Trust, Birmingham, UK

**Keywords:** COVID-19, Vaccine, Adverse events, Autoimmune diseases

## Abstract

Notwithstanding the wealth of literature on COVID-19, studies focusing on young adults with autoimmune diseases (AD) are lacking. To determine early (within 7 days) and late (after 7 days) anti-SARS-CoV-2 vaccine-related adverse events (AEs), post-vaccine disease flares, COVID-19 severity and breakthrough infections (B-INFs) in young people with rheumatic diseases (RMDs) and non-rheumatic autoimmune diseases (nr-ADs) compared to healthy controls (HC). Data were captured through the international COVID-19 vaccination in autoimmune diseases (COVAD) 1 and 2 questionnaires. Of 20,685 complete responses, we identified 6010 from patients aged 18–35 years (1692 RMD, 400 nrADs, 3918 HC) who received up to 4 vaccine doses. BNT162b2 was the most frequently administered vaccine and prior to vaccination, 7% of people with nrAD were taking immunosuppressants (IS) versus 80% in RMDs. Early mild AEs were more frequent in RMDs (93%) and nr-ADs (92%) compared to HC (85%). The frequency of late mild AEs was < 20% in all groups. Severe AEs were rare. SARS-CoV-2 infection rates were similar across all groups, however, RMD patients reported a single episode of infection more frequently than nrADs and HC, while nrADs reported multiple infections more frequently than RMD. Self-reported disease flares were reported by 10% or RMD and 7% of nrAD patients. Our study reinforces the safety of anti-SARS-CoV-2 vaccine also in young people with ADs, but it also highlights that among young individuals the number and clinical picture of SARS-CoV-2 infections is affected more by the type of AD rather than by coexisting IS therapy.

**Supplementary Information:**

The online version contains supplementary material available at 10.1007/s00296-024-05654-w.

## Introduction

Since the beginning of the Severe Acute Respiratory Syndrome CoronaVirus 2 (SARS-CoV-2) pandemic, the scientific literature has exponentially expanded with articles describing the outcome of SARS-CoV-2 infection in healthy individuals and patients with chronic diseases [1]. Likewise, with the marketing of vaccines, physician and patient reported data on their efficacy and safety have been increasingly published [2]. Overall, the available information on SARS-CoV-2 infection and vaccination covers the lifespan with studies being conducted both in the pediatric and the adult population. However, the heterogeneity within either population accounts for the need to conduct targeted studies within specific subpopulations such as toddlers, adolescents or elderly [3]. In this setting, despite the wealth of literature on SARS-CoV-2 infection and vaccination, studies focusing on young adults with autoimmune diseases (AD) are lacking [2]. The term “young patients” has been broadly used in the literature to identify older paediatric populations rather than young adults, hence plenty of data is available in adolescents up to 18 years of age. On the contrary, data on individuals aged above 18 years and up to 40–45 years are often derived from broader cohorts of adults where the proportion of young adults is rather small [4] Young adults with a chronic inflammatory disease are often exposed to prolonged periods of immunosuppression (IS), particularly when diagnosed in childhood, and their health-related outcomes may be worse than those of individuals diagnosed in adulthood [5]. Research has highlighted older age as a risk factor for severe SARS-CoV-2 infection and as a determinant for reduced safety and efficacy of anti-SARS CoV-2 vaccination [2]. However, while the focus on the vulnerable elderly population has been important, this has arguably resulted in an inadequate understanding of the impact of SARS-CoV-2 on the young adult population.

In order to fill this knowledge gap, we aimed to determine early (within 7 days) and late (after 7 days) anti-SARS-CoV-2 vaccine-related adverse events (AEs), post-vaccination/post-infection disease flares, COVID-19 severity and breakthrough infections (B-INFs) in young people with rheumatic diseases (RMDs) and non-rheumatic autoimmune diseases (nr-ADs) compared to healthy controls (HC).

## Methods

We conducted an international, online, cross-sectional survey across 94 Countries focusing on young adults (18–35 years) with ADs and capturing data on vaccination-related AEs and COVID-19 outcomes as a part of the COVAD initiative (Supplementary Figure S1) [6, 7] This questionnaire included questions on demographics, disease specific information such as disease type, duration and symptoms, as well as information on COVID-19 vaccination (type, doses, AEs) and SARS-CoV-2 infection (history, clinical features and complications). Informed consent was obtained electronically through an initial question in the online survey, prior to the main study questionnaire. No incentives were offered for survey completion. Central approval was obtained from the Sanjay Gandhi Postgraduate Institute of Medical Sciences (SGPGIMS) ethics committee as per local guidelines [Institutional Ethics Committee of Sanjay Gandhi Postgraduate Institute of Medical Sciences, Raebareli Road, Lucknow, 226014 (IEC Code: 2021–143-IP-EXP-39)]. The Checklist for Reporting results of the Internet E-Surveys (CHERRIES) was adhered to when reporting results [8]. Additional methods have been detailed in the COVAD study protocol [6, 7]. Responses to open-ended questions were translated using DeepL or Google translate and native speakers were consulted in case of doubts.

Young patients were identified as those aged between 18 and 35 years according to the EULAR Young PARE definition [9] and only responses from individuals within this age range were analysed.

### Statistical analysis

Data analysis was performed with IBM SPSS 28.0 software and variables were compared using the Chi square test, the Mann Whitney U test or the Kruskal Wallis test with multiple post-hoc comparisons. Univariate logistic regression was also performed and variables that differed across groups in univariable analysis were included in multivariable binary logistic regression analysis with adjustment for baseline factors defined a priori (gender, ethnicity and vaccine type). All tests were two-tailed and values of p < 0.05 were considered significant.

## Results

### Study cohort

Of 20,685 respondents who completed the survey, we identified 6010 valid responses from young individuals aged 18–35 years from 97 countries (Supplementary Table S1). The subgroup of patients with RMDs included 1692 individuals, the subgroup of nrADs 400 individuals and the group of HCs 3918 individuals (Table 1). Female gender was slightly more represented in the disease groups while ethnic composition was comparable across all three groups, with Caucasian, Asian and Hispanic ethnicities being the most represented. BNT162b2 was the most frequently administered vaccine in the disease groups whereas Sinopharm was the most frequently administered vaccine in the HC group. Prior to vaccination, only 7% of people with nrAD were taking immunosuppressants (IS) versus 80% of people with RMDs. Likewise, < 5% of people with nrAD were taking glucocorticoids (GC) versus 30% of people with RMDs.

**Table 1:** Characteristics of the study cohort

	RMDs N = 1692	nr-ADs N = 400	HC N = 3918
Age, years, mean ± SD	29 ± 4	28 ± 4	27 ± 4
Disease duration, years, mean ± SD	7.2 ± 6	7.1 ± 5	NA
Number (%) of individuals			
Female gender	1408 (83)	343 (86)	2510 (64)
Ethnicity			
Caucasian (white)	522 (31)	200 (50)	1371 (35)
Asian	637 (38)	86 (21)	1108 (28)
Hispanic	180 (11)	59 (15)	679 (17)
African American or of African origin	106 (6)	6 (1)	73 (2)
Other/do not wish to disclose	247 (14)	49 (12)	687 (17)
N vaccine doses received			
1	313 (18)	102 (25)	652 (17)
2	822 (49)	206 (52)	2498 (64)
3	456 (27)	85 (21)	696 (17)
4	101 (6)	7 (2)	72 (2)
Vaccine type			
BNT162b2 (Pfizer-BioNTech)	263 (15)	95 (24)	719 (18)
ChAdOx1nCoV-19 (Oxford/Astra Zeneca)	85 (5)	29 (7)	180 (5)
JNJ-78436735 (Johnson & Johnson)	13 (0.8)	6 (1.5)	21 (0.5)
mRNA-1273 (Moderna)	65 (4)	14 (3.5)	78 (2)
NVX-CoV2373 (Novavax)	2 (0.1)	1 (0.3)	1 (0.01)
ChAdOx1 nCoV-19 (Covishield)	100 (6)	31 (8)	346 (9)
BBV152 (Covaxin)	23 (1.4)	7 (2)	66 (2)
Gam-COVID-Vac (Sputnik)	10 (0.6)	2 (0.5)	42 (1)
BBIBP-CorV (Sinopharm)	62 (4)	58 (14)	950 (24)
Others/I am not sure	88 (5)	18 (4)	134 (3)
Disease			
Inflammatory arthritis (RA, PsA, SpA)	680 (40)	–	NA
Connective tissue diseases and vasculitis	886 (52)	–	
Other RMD	126 (8)	–	
Endocrine diseases^a^	–	295 (74)	
Haematological diseases	–	15 (4)	
Neurological diseases	–	33 (8)	
Gastrointestinal diseases	–	29 (7)	
Dermatological diseases	–	4 (1)	
Other nrAD	–	24 (6)	
Immunosuppressive therapy prior vaccination	1546 (91)	85 (21)	NA
Glucocorticoids prior vaccination			
Yes, < 10 mg a day	552 (33)	30 (7)	NA
Yes, 10 mg—20 mg a day	150 (9)	6 (1.5)	
Yes, > 20 mg a day	52 (3)	4 (1)	

*RMD* rheumatic and musculoskeletal diseases, *nrADs* non-rheumatic autoimmune diseases, *HC* healthy controls, *RA* rheumatoid arthritis, *PsA* psoriatic arthritis, *SpA* spondyloarthritis, *SD* standard deviation, *NA* not applicable

^a^257 (87%) autoimmune thyroid diseases; 33 (11%) type 1 diabetes; 5 (2%) other endocrine autoimmune diseases

The majority of individuals in each group (RMDs 82%, nrADs 75%, HC 83%) had received ≥ 2 vaccine doses at the time of survey completion. The analysis revealed patterns in vaccine-related adverse events, disease flares, COVID-19 severity, and breakthrough infections among patients with rheumatic diseases (RMDs), non-rheumatic ADs (nr-ADs), and healthy controls (HCs).

### AEs in individuals having received 1 and/or 2 doses

Early mild AEs were more frequent in RMDs (93%) and nr-ADs (92%) compared to HC (85%) (Table 2). Injection site pain, headache, fatigue and body ache were the most frequent mild AE reported in all three groups; however they were more frequent in nr-AD than in HC and RMD (Supplementary Table 2). Fever and chills were also more frequent in nr-AD compared to HC and to RMD.

**Table 2:** Comparison of study subgroups with regard to occurrence of adverse events

	RMDs vs nrADs	RMDs vs HC	nrADs vs HC
	Adjusted OR (95% CI)		
After 1 or 2 vaccine doses			
Any early mild AE (< 7 days)	1.1 (0.7–1.6)	2.4 (2.0–3.1)	2.0 (1.4–2.9)
Any late mild AE (> 7 days)	0.8 (0.6–1.2)	0.7 (0.3–1.3)	1.2 (0.6–2.4)
Any severe AE	0.4 (0.2–1.2)	1.7 (0.7–3.8)	2.7 (0.9–8.2)
After 3 or 4 vaccine doses			
Any early mild AE (< 7 days)	–	–	–
Any late mild AE (> 7 days)	0.9 (0.5–1.6)	1.0 (0.8–1.3)	1.1 (0.6–1.8)
Any severe AE after the 3rd dose	0.7 (0.2–2.5)	1.6 (0.7–3.7)	2.3 (0.6–8.4)
Any severe AE after the 4th dose	–	–	–

*RMD* rheumatic and musculoskeletal diseases, *nrADs* non-rheumatic autoimmune diseases, *HC* healthy controls, *AE* adverse events, *OR* odds ratio, *CI* confidence interval

The frequency of late mild AEs after the 1st and 2nd dose was low and similar across all three groups (after 1st dose: RMD 2.9%, nrADs 2.0%; HC 2.4%; After 2nd dose: RMDs 7.6%, nrADs 6.6%; HC 6.6%). (Table 2). Severe AEs were rare (< 2%) in all groups, with no differences between the first and second vaccine dose.

### AEs in individuals having received 3 or 4 doses

The frequency of late mild AEs after any dose was < 20% in all three groups with no significant differences observed upon comparison (RMD 18–21%, nr-AD 14–18% HC 14–19%) (Table 2). Severe AEs after the third dose were rare (RMD 2.2–3% nr-AD 0–3.5% HC 1.1–4%). In the 1417 subjects that received a fourth dose, only two AEs (0.1%) were reported, one in a subject with RMD and one in a HC.

### Pre-vaccine vs breakthrough infections (B-INFs)

The frequency of reported SARS-CoV-2 infections was similar in all three groups (RMD = 28%, nr-ADs = 25%, HC = 28%) and overall severe infections (i.e., requiring hospitalisation or oxygen therapy) were rarely reported (Table 3). However, patients with RMDs reported experiencing a single episode of infection more frequently than nrADs and HC, while nrADs reported experiencing multiple infections more frequently than RMD. The frequency of pre-vaccine infections was lower in RMD compared to HC (OR 0.6, 95% CI 0.4–0.9), while it was similar in nr-AD vs HC. In contrast, the frequency of B-INFs was higher in RMD vs HC (OR 2.7 95% CI 2.1–3.5), while it was similar in nr-AD vs HC. Regarding the clinical picture of pre-vaccine infections versus B-INFs no differences were observed among respondents with nrADs (Supplementary Table S3). Conversely, a significantly lower frequency of loss of smell and loss of taste and a significantly higher frequency of running nose was reported for B-INFs compared to pre-vaccine infections by both patients with RMD and HC. HC also reported less frequently skin rashes during B-INFs compared with pre-vaccine infections. Given that less than 5% of respondents received advanced therapies for SARS-CoV-2 infection, it is ulikely that the observed shifts in clinical presentation can be attributed to different infection management strategies.

**Table 3:** SARS-CoV-2 infection history and comparison across the study subgroups

	RMDs N = 1692	nr-ADs N = 400	HC N = 3918
	N (%)		
Ever tested positive for SARS-CoV2	469 (28)	98 (24)	1095 (28)

	RMDs N = 469	nr-ADs N = 98	HC N = 1095
	N (%)		
Asymptomatic infection	13 (3)	0 (0)	38 (3)
Required hospital admission	21 (4)	2 (2)	11 (1)
Required oxygen therapy	9 (2)	1 (1)	7 (1)
Required ICU admission	3 (0.6)	0 (0)	2 (0.2%)

	RMDs vs nr-Ads	RMDs vs HC	nrADs vs HC
	Adjusted OR (95% CI)		
SARS CoV-2 infection timing			
Before vaccination	1.1 (0.6–1.8)	0.6 (0.4–0.9)	0.9 (0.6–1.5)
After vaccination (B-INFs)	1.4 (0.9–2.2)	2.7 (2.1–3.5)	1.5 (0.9–2.3)
SARS CoV-2 infection frequency			
Once	0.9 (0.6–1.3)	3.3 (2.8–3.9)	1.0 (0.7–1.4)
Twice or more	0.4 (0.2–0.8)	0.5 (0.4–0.8)	1.3 (0.7–2.2)

*RMD* rheumatic and musculoskeletal diseases, *nrADs* non-rheumatic autoimmune diseases, *HC* healthy controls, *AE* adverse events, *OR* odds ratio, *CI* confidence interval, *B-INF* breakthrough infections

Subsequently we compared the clinical picture of pre-vaccine and B-INFs between the groups (Supplementary Table S4). Arthralgia was more frequent in RMD patients compared to HC both for pre-vaccine infections and for B-INFs. Conversely cough and headache were more prevalent in RMDs compared to HC in pre-vaccine infections but were reported by a similar number of respondents with RMD and HC during B-INFs. Nausea and vomiting were reported more frequently by patients with RMD compared to HC both for pre-vaccine infections and for in B-INFs, although the OR for B-INFs was lower. Cough was also more prevalent in patients with nrADs compared to HC in pre-vaccine infections but was reported by a similar number of patients with nrADs and HC during B-INFs. Skin rashes were reported by a similar number of patients with nrADs and HC during pre-vaccine infections but were significantly more frequent in patients with nrADs than in HC during B-INFs.

### Post-vaccination and post-infection disease flares

Self-reported disease flares after the second vaccine dose were reported by 10% of patients with RMDs and 7% of patients with nrAD (OR 1.7, 95% CI 1.1–2.7). Of these, 41% of RMDs and 27% or nr-ADs reported that this required a change in their medications (increased dose/addition of a new IS and/or GC)). Self-reported disease flares after SARS-CoV2 infection were reported by 5% of patients with RMDs and 1.5% of patients with nrADs.

## Discussion

Our study addressed a critical gap in the literature, offering a detailed exploration of SARS-CoV-2 vaccination and infection outcomes in young adults with ADs and providing insights into the impact of disease type on these outcomes. With regards to SARS-CoV-2 infection in patients with RMDs, a systematic literature review conducted to inform EULAR recommendations indicated that patients with RMDs do not have an increased risk of contracting SARS-CoV-2 infection compared to HCs [2]. However, it is important to note that these conclusions were drawn from data collected during the early phases of the pandemic when vaccines were not widely available, a period characterised by stringent shielding policies for people with chronic diseases and those on immunosuppression, which could have mitigated infection rates in these groups [10]. This is consistent with our results demonstrating a lower frequency of pre-vaccine infections in respondents with RMD than in HCs. Conversely, young patients with nrADs were significantly less exposed to IS agents and were subject to shielding policies widely varying across the different conditions [11, 12]. This may explain our finding of a similar frequency of pre-vaccine infections in patients with nr-AD compared to HC.

The association between advancing age and adverse COVID-19 outcomes has been well-documented, both in the general population and in those with chronic disease such as RMDs [2]. However, this association was observed in earlier phases of the pandemic when SARS-CoV-2 infection occurred in non-vaccinated or partially vaccinated individuals. More recent studies exploring B-INFs indicates that age is no longer a consistent predictor of infection risk or adverse outcomes in patients with RMDs [13].

Interestingly, despite less exposure to GC and IS, respondents to our survey with nr-AD reported an overall higher number of SARS-CoV-2 infections compared to those with RMD. No difference in the clinical picture of pre-vaccine infections vs B-INFs but a more pronounced burden of post-vaccine mild AEs was observed in nr-AD compared to RMD. These findings may highlight that the type of autoimmune disease rather than the degree of IS may play a more crucial role in determining vaccine safety, infection risk and the characteristics of B-INFs in young people. Less than 5% of respondents received advanced therapies for SARS-CoV-2 infection (e.g., monoclonal antibodies or antivirals) hence the differences (or lack thereof) in the clinical pictures are not biased by different therapeutic approaches throughout different phases of the pandemic.

Reassuringly, the occurrence of post-infection and post-vaccination disease flares was low, affecting less than 10% of patients. However, patients with RMDs had a higher risk of flares compared to nr-ADs and this was more evident for post-vaccination flares. In our cohort the majority of nr-AD patients suffered with thyroid disease and our findings are in line with those of a systematic review reporting very few cases of post-infection thyroid disease flares [14].

Our study displays some limitations that need to be acknowledged. First, self-reported data presents difficulties due to potential memory and selection bias and lack of verification through medical records may affect accuracy. Furthermore, although the study encompasses a wide demographic, certain subgroups may be underrepresented. However, our study also has the strength to have filled a knowledge gap with data from diverse regions and extensive participant engagement, and contributes valuable information to the understanding of COVID-19 dynamics in AD populations.

In conclusion, the findings of the present study add an important piece of information to existing body of literature demonstrating that in young people the type of disease (rheumatic vs non rheumatic) rather than coexisting immunosuppressive treatment seems the key variable in influencing outcomes of SARS-CoV-2 infection and vaccination. This highlights the need for tailored strategies for vaccination and monitoring of infections.

### Supplementary Information

Below is the link to the electronic supplementary material.

Supplementary file1 (DOCX 725 KB)

Supplementary file2 (DOCX 23 KB)

## Data Availability

The datasets generated and/or analyzed during the current study are not publicly available but are available from the corresponding author upon reasonable request.
