# Supplementary material for: COVID-19 severity, breakthrough infections and vaccine safety in young individuals with autoimmune diseases: insights from the COVAD study
Source: Rheumatol Int. 2024 Jul 13;44(9):1725–31. doi: 10.1007/s00296-024-05654-w (PMC11343807; doi:10.1007/s00296-024-05654-w)
Supplement: Supplementary file 1 — Supplementary file1 (DOCX 725 KB) [file 296_2024_5654_MOESM1_ESM.docx]

**Supplementary Figure S1**. Map showing the 94 Countries where the survey was distributed by the COVAD Study Group


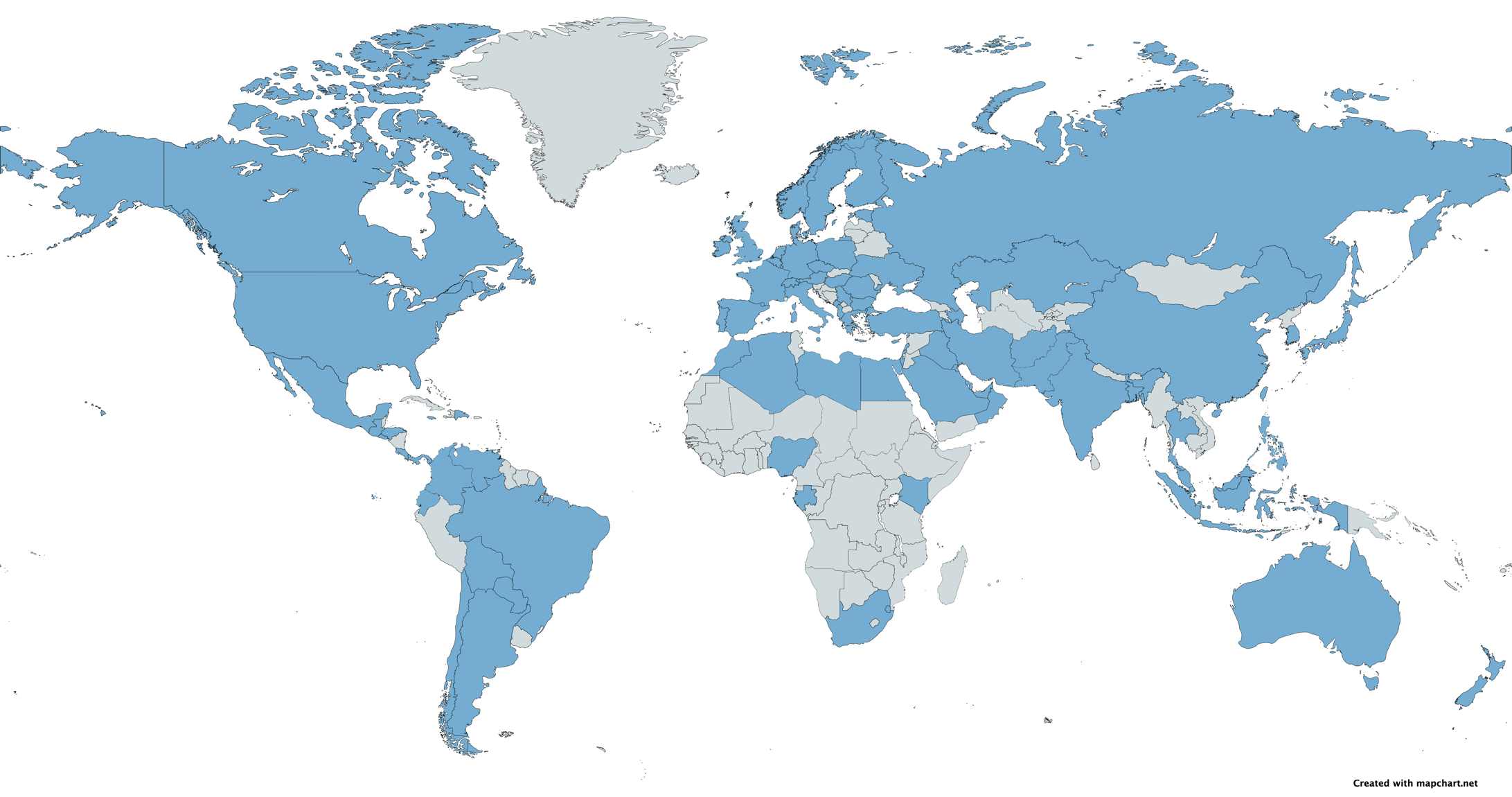


**Supplementary Table S1** Number of survey respondents by Country (N=6010, 97 Countries)

| **Country** | **Number** | **%** | **Country** | **Number** | **%** |
| --- | --- | --- | --- | --- | --- |
| Turkey | 1182 | 19.7 | Mauritius | 8 | 0.1 |
| India | 877 | 14.6 | Austria | 7 | 0.1 |
| Mexico | 817 | 13.6 | Bahrain | 7 | 0.1 |
| Poland | 162 | 2.7 | China | 6 | 0.1 |
| Philippines | 150 | 2.5 | Hungary | 6 | 0.1 |
| Pakistan | 146 | 2.4 | Oman | 6 | 0.1 |
| United Kingdom | 129 | 2.1 | Australia | 5 | 0.1 |
| Italy | 125 | 2.1 | El Salvador | 5 | 0.1 |
| Thailand | 124 | 2.1 | Kuwait | 5 | 0.1 |
| Bulgaria | 118 | 2 | Algeria | 4 | 0.1 |
| Brazil | 115 | 1.9 | Ethiopia | 4 | 0.1 |
| Bangladesh | 114 | 1.9 | Qatar | 4 | 0.1 |
| Peru | 110 | 1.8 | Ukraine | 4 | 0.1 |
| Egypt | 105 | 1.7 | Afghanistan | 3 | 0.0 |
| United States | 97 | 1.6 | Honduras | 3 | 0.0 |
| Malaysia | 83 | 1.4 | Kazakhstan | 3 | 0.0 |
| Lebanon | 81 | 1.3 | Netherlands | 3 | 0.0 |
| United Kingdom | 79 | 1.3 | Nicaragua | 3 | 0.0 |
| Saudi Arabia | 76 | 1.3 | Portugal | 3 | 0.0 |
| Guatemala | 73 | 1.2 | Romania | 3 | 0.0 |
| Indonesia | 73 | 1.2 | Belgium | 2 | 0.0 |
| Colombia | 70 | 1.2 | Cyprus | 2 | 0.0 |
| Russian Federation | 68 | 1.1 | Czech Republic | 2 | 0.0 |
| United States of America | 63 | 1 | Haiti | 2 | 0.0 |
| Ghana | 58 | 1 | Ireland {Republic} | 2 | 0.0 |
| Sweden | 57 | 0.9 | Libya | 2 | 0.0 |
| Switzerland | 57 | 0.9 | Liechtenstein | 2 | 0.0 |
| Nepal | 56 | 0.9 | Serbia | 2 | 0.0 |
| Germany | 53 | 0.9 | Albania | 1 | 0.0 |
| Spain | 52 | 0.9 | Andorra | 1 | 0.0 |
| Israel | 50 | 0.8 | Angola | 1 | 0.0 |
| Nigeria | 47 | 0.8 | Armenia | 1 | 0.0 |
| Japan | 43 | 0.7 | Azerbaijan | 1 | 0.0 |
| Taiwan | 42 | 0.7 | Burkina Faso | 1 | 0.0 |
| Venezuela | 36 | 0.6 | Cameroon | 1 | 0.0 |
| United Arab Emirates | 34 | 0.6 | Gabon | 1 | 0.0 |
| Argentina | 31 | 0.5 | Greece | 1 | 0.0 |
| Ecuador | 31 | 0.5 | Grenada | 1 | 0.0 |
| Morocco | 30 | 0.5 | Iran | 1 | 0.0 |
| France | 24 | 0.4 | Jamaica | 1 | 0.0 |
| Iraq | 21 | 0.3 | Kenya | 1 | 0.0 |
| Chile | 19 | 0.3 | Korea South | 1 | 0.0 |
| Costa Rica | 19 | 0.3 | Kyrgyzstan | 1 | 0.0 |
| Canada | 17 | 0.3 | Malawi | 1 | 0.0 |
| Panama | 16 | 0.3 | Montenegro | 1 | 0.0 |
| Bolivia | 14 | 0.2 | Somalia | 1 | 0.0 |
| Dominican Republic | 14 | 0.2 | Tuvalu | 1 | 0.0 |
| Jordan | 12 | 0.2 | Uzbekistan | 1 | 0.0 |
| Paraguay | 12 | 0.2 |  |  |  |

**Supplementary Table S2** Frequency of early mild adverse events in the study subgroups

| **Early mild AE** | **RMDs N=1135** | **nrADs N=308** | **HC N=3150** |
| --- | --- | --- | --- |
|  | **Number (%) of individuals** | | |
| Injection site (arm) pain and soreness | 516 (45) | 194 (63) | 1661 (53) |
| Muscle pain in all arms and legs | 125 (11) | 49 (16) | 410 (13) |
| Body ache | 199 (17) | 76 (25) | 555 (18) |
| Fever | 187 (16) | 69 (22) | 525 (17) |
| Chills | 117 (10) | 28 (19) | 319 (10) |
| Nausea/Vomiting | 47 (4) | 18 (6) | 130 (4) |
| Headache | 218 (19) | 81 (26) | 602 (19) |
| Rash | 13 (1) | 2 (1) | 21 (1) |
| Fatigue | 244 (21) | 95 (31) | 704 (22) |
| Diarrhoea | 21 (2) | 9 (3) | 74 (2) |
| Abdominal pain | 20 (2) | 8 (3) | 34 (1) |
| High pulse rate or palpitations | 27 (2) | 19 (6) | 66 (2) |
| Rise in blood pressure | 3 (0.3) | 6 (2) | 10 (0.6) |
| Fainting | 3 (0.3) | 1 (0.3) | 14 (0.4) |
| Difficulty in breathing | 14 (1.2) | 8 (2.6) | 23 (0.7) |
| Dizziness | 54 (5) | 19 (6) | 128 (4) |
| Chest pain | 10 (1) | 6 (2) | 29 (1) |
| *RMD, rheumatic and musculoskeletal diseases; nrADs, non-rheumatic autoimmune diseases; HC, healthy controls* | | | |

**Supplementary Table S3** Comparison of the clinical picture of SARS-CoV-2 infection within the study subgroups

|  | **B-INF vs pre-vaccine infection** | | |
| --- | --- | --- | --- |
|  | **RMDs** | **nrADs** | **HC** |
|  | **Adjusted OR (95% CI)** | | |
| **Fever** | 0.7 (0.4-1.2) | 0.9 (0.3-3.0) | 0.9 (0.6-1.3) |
| **Fatigue** | 1.2 (0.7-2.0) | 0.8 (0.2-2.6) | 1.1 (0.7-1.5) |
| **Myalgia** | 0.9 (0.6-1.5) | 0.6 (0.2-2.0) | 1.1 (0.7-1.5) |
| **Arthralgia** | 0.9 (0.5-1.4) | 1.2 (0.3-5.4) | 0.8 (0.6-1.3) |
| **Cough** | 1.5 (0.9-2.5) | 0.4 (0.1-1.4) | **1.9 (1.3-2.7)** |
| **Shortness of breath** | 1.1 (0.6-2.0) | 0.4 (0.1-1.6) | **0.6 (0.4-0.9)** |
| **Loss of smell** | **0.3 (0.2-0.5)** | 0.4 (0.1-1.4) | **0.3 (0.2-0.5)** |
| **Loss of taste** | **0.3 (0.2-0.5)** | 0.4 (0.1-1.4) | **0.3 (0.2-0.4)** |
| **Running nose** | **2.1 (1.2-3.5)** | 0.8 (0.2-2.7) | **1.7 (1.2-2.6)** |
| **Congestion** | 1.7 (0.9-3.0) | 0.7 (0.2-2.6) | 1.2 (0.8-1.8) |
| **Throat pain** | 1.7 (0.9-3.8) | 0.9 (0.3-3.0) | **1.7 (1.1-2.4)** |
| **Chest pain** | 0.8 (0.4-1.5) | 0.3 (0.1-1.9) | 0.7 (0.4-1.2) |
| **Diarrhoea** | 0.6 (0.3-1.1) | 1.2 (0.2-7.0) | 1.0 (0.6-1.7) |
| **Headache** | 1.1 (0.7-1.8) | 0.3 (0.1-1.0) | 0.8 (0.6-1.2) |
| **Oral ulcers** | 1.0 (0.3-3.9) | 0.4 (0.02-6.5) | 2.3 (0.3-21.4) |
| **Nausea/vomiting** | 0.8 (0.4-1.6) | 1.2 (0.1-12.5) | 1.5 (0.7-3.4) |
| **Abdominal pain** | 1.3 (0.6-2.9) | 0.8 (0.1-9.3) | 1.8 (0.8-3.8) |
| **Skin rashes** | 0.8 (0.3-2.4) | 0.8 (0.1-9.3) | **0.2 (0.03-0.8)** |
| *RMD, rheumatic and musculoskeletal diseases; nrADs, non-rheumatic autoimmune diseases; HC, healthy controls; OR, odds ratio; CI, confidence interval; B-INF, breakthrough infections* | | | |

**Supplementary Table S4** Comparison of the clinical picture of SARS-CoV-2 pre vaccine and post vaccine infection between study subgroups

|  | **RMDs vs HC** | | **nrADs vs HC** | |
| --- | --- | --- | --- | --- |
|  | **Adjusted OR (95% CI)** | | | |
|  | **Pre vaccine infection** | **B-INFs** | **Pre vaccine infection** | **B-INFs** |
| Fever | 1.3 (0.8-2.2) | 1.5 (0.9-2.0) | 0.6 (0.2-1.9) | 0.7 (0.3-1.3) |
| Fatigue | 0.9 (0.6-1.6) | 1.1 (0.8-1.6) | 0.9 (0.3-2.6) | 0.7 (0.3-1.3) |
| Myalgia | 1.0 (0.6-1.7) | 0.9 (0.6-1.2) | 1.6 (0.5-4.6) | 0.9 (0.5-1.8) |
| Arthralgia | **2.2 (1.2-3.6)** | **2.2 (1.5-3.1)** | 0.7 (0.2-2.5) | 1.0 (0.4-2.1) |
| Cough | **1.7 (1.1-2.9)** | 1.4 (0.9-1.99) | **3.3 (1.1-10.2)** | 0.7 (0.4-1.4) |
| Shortness of breath | 1.1 (0.6-2.0) | 1.5 (0.9-2.4) | 2.3 (0.8-7.0) | 1.8 (0.8-4.1) |
| Loss of smell | 1.0 (0.6-1.7) | 0.9 (0.6-1.4) | 0.9 (0.3-2.7) | 1.1 (0.5-2.3) |
| Loss of taste | 0.9 (0.5-1.5) | 1 (0.7-1.6) | 0.6 (0.2-1.9) | 0.9 (0.4-2.0) |
| Running nose | 1.2 (0.7-2.1) | 1.5 (1.0-2.0) | 2.0 (0.7-6.0) | 0.9 (0.5-1.9) |
| Congestion | 0.9 (0.5-1.7) | 1.4 (0.9-1.9) | 1.2 (0.4-3.9) | 0.7 (0.3-1.6) |
| Throat pain | 1.3 (0.8-2.3) | 1.4 (0.9-1.9) | 2.0 (0.7-5.9) | 1.1 (0.6-2.2) |
| Chest pain | 1.5 (0.8-3.1) | 1.6 (0.9-2.7) | 1.5 (0.4-5.9) | 0.8 (0.2-2.7) |
| Diarrhoea | **2.2 (1.2-4.3)** | 1.4 (0.9-2.2) | 0.9 (0.2-4.7) | 1.2 (0.5-3.1) |
| Headache | 1.2 (0.7-1.9) | **1.6 (1.2-2.3)** | 2.1 (0.7-6.3) | 0.7 (0.3-1.4) |
| Oral ulcers | 6.6 (0.7-64.0) | 2.8 (0.2-2.3) | 13.3 (0.8-224) | 2.1 (0.2-19.0) |
| Nausea/vomiting | **4.7 (2.0-11.1)** | **2.5 (1.5-4.5)** | 1.4 (0.2-12) | 1.1 (0.3-3.8) |
| Abdominal pain | 2.2 (0.9-5.9) | 1.7 (0.9-2.9) | 3.0 (0.6-11.5) | 0.3 (0.03-2.3) |
| Skin rashes | 1.5 (0.5-5.0) | 4.2 (0.8-21.0) | 1.8 (0.2-16.0) | **8.7 (1.2-64)** |
| *RMD, rheumatic and musculoskeletal diseases; nrADs, non-rheumatic autoimmune diseases; HC, healthy controls; AE, adverse events; OR, odds ratio; CI, confidence interval; B-INF, breakthrough infections* | | | | |
